# Pronounced Structural and Functional Damage in Early Adult Pediatric-Onset Multiple Sclerosis with No or Minimal Clinical Disability

**DOI:** 10.3389/fneur.2017.00608

**Published:** 2017-11-14

**Authors:** Antonio Giorgio, Jian Zhang, Maria Laura Stromillo, Francesca Rossi, Marco Battaglini, Lucia Nichelli, Marzia Mortilla, Emilio Portaccio, Bahia Hakiki, Maria Pia Amato, Nicola De Stefano

**Affiliations:** ^1^Department of Medicine, Surgery and Neuroscience, University of Siena, Siena, Italy; ^2^Anna Meyer Children’s University Hospital, Florence, Italy; ^3^IRCCS Don Gnocchi Foundation, Florence, Italy; ^4^Department of Neurology, University of Florence, Florence, Italy

**Keywords:** multiple sclerosis, MRI, disability, diffusion tensor imaging, resting state networks, connectivity, atrophy

## Abstract

Pediatric-onset multiple sclerosis (POMS) may represent a model of vulnerability to damage occurring during a period of active maturation of the human brain. Whereas adaptive mechanisms seem to take place in the POMS brain in the short-medium term, natural history studies have shown that these patients reach irreversible disability, despite slower progression, at a significantly younger age than adult-onset MS (AOMS) patients. We tested for the first time whether significant brain alterations already occurred in POMS patients in their early adulthood and with no or minimal disability (*n* = 15) in comparison with age- and disability-matched AOMS patients (*n* = 14) and to normal controls (NC, *n* = 20). We used a multimodal MRI approach by modeling, using FSL, voxelwise measures of microstructural integrity of white matter tracts and gray matter volumes with those of intra- and internetwork functional connectivity (FC) (analysis of variance, *p* ≤ 0.01, corrected for multiple comparisons across space). POMS patients showed, when compared with both NC and AOMS patients, altered measures of diffusion tensor imaging (reduced fractional anisotropy and/or increased diffusivities) and higher probability of lesion occurrence in a clinically eloquent region for physical disability such as the posterior corona radiata. In addition, POMS patients showed, compared with the other two groups, reduced long-range FC, assessed from resting functional MRI, between default mode network and secondary visual network, whose interaction subserves important cognitive functions such as spatial attention and visual learning. Overall, this pattern of structural damage and brain connectivity disruption in early adult POMS patients with no or minimal clinical disability might explain their unfavorable clinical outcome in the long term.

## Introduction

Plasticity is an essential asset of the human brain, mainly occurring during development and being able to dynamically act in response to environment and personal experiences through shaping of neural connections. Whereas plasticity is beneficial during normal development by means of adaptive changes, little is known in the setting of brain damage during developmental age.

Multiple sclerosis (MS) is the main cause of non-traumatic disability in young adulthood in Western Countries. Pediatric-onset MS (POMS) (i.e., with the first MS clinical attack before 18 years of age) characterizes up to 10% of all MS patients (1). POMS may thus represent a model of vulnerability to damage occurring during active brain maturation.

Recently, various studies using different MRI techniques sought to define the underlying pathology of POMS patients during childhood and/or adolescence and thus in the short-medium term. Indeed, diffusion tensor imaging (DTI) studies using region-of-interest and tract-based analyses found altered diffusion measures in both visible white matter (WM) lesions and normal-appearing WM (NAWM) of the major WM pathways in POMS but not in pediatric patients with clinically isolated syndrome (2, 3). Recently, using ultrahigh-field 7 T MRI a great number of cortical lesions was identified in a small group of adolescents and young adults with POMS despite relatively short disease duration and limited physical disability, suggesting early cortical gray matter (GM) involvement (4). Some functional MRI (fMRI) studies found no change in effective connectivity of the sensorimotor network (5) and a relative preservation of global internetwork functional connectivity (FC) (6), suggesting that preservation of brain adaptive properties may explain the favorable clinical outcome in the short-medium term. Moreover, it was demonstrated in late adolescent POMS (7) widespread abnormalities of anatomical connectivity (AC), in terms of DTI-derived measures of WM microstructure, and associated higher FC in the default mode network (DMN) and frontoparietal network, with a potential compensatory role.

On the other hand, natural history (8, 9) and cognitive (10) studies showed that POMS patients reach irreversible physical and cognitive disability as well as progressive course at a significantly younger age than adult-onset MS (AOMS) patients. Finally, neuropathology has demonstrated overall higher degree of axon damage in POMS than in AOMS (11).

It is currently unknown whether POMS in the early adulthood and yet without relevant disability can already show pronounced brain tissue damage and how the brain damage during a period of maturation can be translated in terms of disrupted connectivity. For this reason, we assessed macroscopic structural damage as well as integrity of AC and FC for the first time in the brain of early adults with POMS who had no or minimal physical and cognitive disability and compared them with age- and disability-matched AOMS patients and with normal controls (NC).

## Materials and Methods

### Study Subjects

Forty-nine subjects were included in the study, divided into early adult POMS (*n* = 15, age = 24.8 ± 7 years, nine females, disease duration = 9.7 ± 6.2 years, age at onset <18 years (12), median expanded disability status scale [EDSS] (13) = 1 [values: 0, *n* = 2; 1, *n* = 7; 1.5, *n* = 2; 2, *n* = 2; 3, *n* = 1; 3.5, *n* = 1]), AOMS (*n* = 14, age = 27.8 ± 3 years, 10 females, disease duration = 5 ± 2.7 years, median EDSS = 1 [values: 0, *n* = 1; 1, *n* = 8; 1.5, *n* = 1; 2, *n* = 2; 3, *n* = 1; 3.5, *n* = 1]), and NC (*n* = 20, 15 females, age = 27.1 ± 4 years). To be included, MS patients had to be relapse- and steroid treatment-free for at least 3 months. Cognition was assessed by a trained neuropsychologist, blinded to the clinical/MR data, in 11 of 15 POMS and 11 of 14 AOMS using the Brief Repeatable Battery (BRB) of neuropsychological tests (14, 15). The performance on each BRB test was assessed by applying Italian normative values (16). Failure of a test was defined when the score was below the 10th percentile, to capture even subtle cognitive impairment.

Normal control group was recruited among laboratory and hospital workers, had normal neurological examination and no history of neurological disorder.

The study received approval from the local Ethics Committee (Azienda Ospedaliera Universitaria Senese). Informed written consent was obtained from all subjects in accordance with the Declaration of Helsinki before study entry.

### MRI Acquisition

Brain MRI was acquired on a 3 T Philips scanner (Philips Medical Systems, Best, The Netherlands) located at Meyer University Hospital, Florence. A sagittal survey image was used to identify the anterior and posterior commissures. Sequences were acquired in the axial plane parallel to the bicommissural line. A dual-echo, turbo spin-echo sequence [repetition time (TR)/echo time (TE)1/TE2 = 4,000/10/100 ms, voxel size = 1 mm × 1 mm × 3 mm] yielded proton density (PD) and T2-weighted (T2-W) images. DTI data consisted of echo-planar imaging (EPI) (TR = 7,036 ms; TE = 196 ms; voxel size = 2.5 mm^3^), with 32 diffusion directions and *b*-value = 900 s/mm^2^. The resting-fMRI data were 200 volumes of EPI sequence with TR = 3,000 ms, TE = 35 ms, voxel size = 1.87 mm × 1.87 mm × 4 mm. A high-resolution (3D) T1-weighted image (T1-W, TR = 10 ms, TE = 4 ms, voxel size = 1 mm^3^) was also acquired for image registration, anatomical mapping and analysis of GM volume.

### MRI Analysis

It was performed at the Quantitative Neuroimaging Laboratory of the University of Siena.

#### Macroscopic Brain Findings: Lesions and Volumes

All MR scans were first visually assessed to rule out the presence of artifacts. Then a single observer, blinded to subject identity, outlined in both MS groups the WM lesions on PD images, also considering information from T2-W images, by using a semiautomated segmentation technique based on user-supervised local thresholding (Jim 5.0,^1^ Xinapse System, Leicester, UK).

Lesion volume (LV) was computed by multiplying lesion area by slice thickness. Lesion probability maps (LPMs) were created for both MS groups using tools of the FMRIB Software Library [FSL^2^ (17, 18)]. First, hypointense WM lesions in 3D T1-W images were filled with intensities of the surrounding NAWM (19), to avoid GM/WM misclassification. Second, a symmetric study-specific template was obtained after registering a sample of T1-W images (*n* = 10 age-matched patients for each MS group) on the high-resolution (1 mm^3^) MNI152 standard image by using first linear [FMRIB Linear Image Registration Tool (FLIRT)] (20), then refinement with non-linear [FMRIB Non-linear Image Registration Tool (FNIRT)] registration (21) and, finally, after averaging all the resulting registered images. Third, the T2-lesion mask of each patient was registered on the template through FLIRT followed by FNIRT (with nearest neighbor interpolation) using the matrices obtained from previous registrations [i.e., PD on the T1-W image with “boundary-based registration” (BBR) cost function, T1-W image on the template]. Two observers (Antonio Giorgio and Jian Zhang) independently checked all the registered T2-lesion masks on the template and an agreement was found in all cases. Fourth, for each MS patient group, LPM was generated by first merging and then averaging all the T2-lesion masks previously registered on the template. The voxel intensity of LPM represents the frequency of lesion occurrence in that voxel. Finally, voxelwise group comparison between LPMs of POMS and AOMS was performed (see Statistics).

Brain volumes were obtained from 3D T1-W images (“lesion filled” in MS patients) with tools of FSL such as Structural Image Evaluation of Normalized Atrophy (SIENAx) which computes volumes of whole brain, WM, GM, and neocortical GM (22) and FMRIB’s Integrated Registration and Segmentation Tool (FIRST) which computes deep GM volumes (23).

Briefly, SIENAX first calls Brain Extraction Tool (BET) (24), which strips non-brain tissue and then uses the brain and skull images to estimate the scaling between the subject’s image and MNI standard space. Later, it runs tissue segmentation with FMRIB Automated Segmentation Tool (25), which estimates the volume of brain tissues, and then it multiplies this by the estimated scaling factor, to account for head-size-related variability across study subjects.

FMRIB’s Integrated Registration and Segmentation Tool is a model-based segmentation/registration tool, in which the shape/appearance models, based on multivariate Gaussian assumptions are constructed from predefined manually segmented images. This tool is able to segment the deep GM, producing mesh and volumetric outputs (applying boundary correction).

#### Voxelwise Analysis of DTI Images

Diffusion tensor imaging data were preprocessed through automatic quality control performed with DTIPrep,^3^ a tool that minimizes various types of artifacts (26). Then, analysis was performed across the whole brain with FSL tools. First, DTI data were corrected for MRI eddy currents and head motion using affine registration to a reference volume, i.e., the one without diffusion weighting (*b* = 0). Second, images of fractional anisotropy (FA), axial diffusivity (AD) and radial diffusivity (RD) were created by fitting a tensor model to the raw DTI data using FMRIB Diffusion Toolbox (27), and then brain extracted using BET. Then we used tract-based spatial statistics (TBSS) (28) for voxelwise analysis of DTI images. In particular, FA images from all subjects were first aligned to the FMRIB58_FA standard-space image (i.e., a high-resolution average of 58 well-aligned good quality FA images from healthy subjects) with FNIRT and then the mean FA image was thinned to create a mean FA “skeleton” (thresholded at FA > 0.2), which represents the centers of all WM tracts common to all study subjects. Aligned FA images were then projected onto this WM skeleton. TBSS was also applied to AD and RD images by using non-linear registration, skeletonization and projection stages from the analysis of FA images. The resulting projected (onto the mean WM skeleton) images of FA, AD, and RD images were finally fed into voxelwise group statistics (see Statistics).

#### Voxelwise Analysis of Resting fMRI

Various preprocessing steps were performed for each resting-fMRI image: removal of the first five volumes to allow signal stability; initial motion correction by volume-realignment to the middle volume using linear registration MCFLIRT (20); non-brain removal using BET; global 4D mean intensity normalization; spatial smoothing (6 mm FWHM); registration to the T1-weighted image using the affine BBR cost function of FLIRT, and subsequent transformation to MNI152 standard space using non-linear registration FNIRT (warp resolution: 10 mm); use of independent component analysis-based automatic removal of motion artifacts^4^ to minimize motion-related artifacts (29); regression of WM and cerebrospinal fluid (both thresholded at a very conservative threshold of 95% tissue probability) to remove residual structured noise; application of a high-pass temporal filtering (cutoff frequency = 100 s); final normalization to MNI152 standard space using FNIRT. The filtered, normalized fMRI images of all study subjects were concatenated across time into a single 4D image, which was then automatically decomposed by Multivariate Exploratory Linear Optimized Decomposition into Independent Components (ICs)^5^ into a set of 27 ICs (30, 31). Finally, voxelwise intranetwork (short-range) FC analysis was performed using the “dual-regression” approach (32), following a previously described procedure (33). The outputs of the first-stage dual regression, i.e., the subject-specific timeseries, were used for estimating temporal correlation between all the RSNs pairs, which is a measure of internetwork (or long-range) FC strength, using FSLNets.^6^ All internetwork Pearson correlation coefficients were transformed into *z*-scores using the Fisher transform to improve data normality and then clustered hierarchically for each of the three group comparisons, thus leading to different functional groupings of the RSNs. Full correlation allows for the influence of other networks on RSN pairs while partial correlation represents a more direct relationship between RSN pairs.

### Statistics

Differences in age and disease duration among the study groups were performed with analysis of variance (ANOVA) using SPSS (v20, IBM).

Voxelwise group differences across the whole brain were performed in the general linear model framework with unpaired *t*-tests (POMS vs AOMS) for LPM and with ANOVA (*F*-test followed by *post hoc* pair comparisons of POMS, AOMS and NC) for AC and FC using “randomize” (34), a nonparametric permutation testing (*n* = 5,000 permutations). Thresholding of statistical images was performed with Threshold-Free Cluster Enhancement, with a significance level of *p* ≤ 0.01, corrected for multiple comparisons across space and with cluster size ≥ 10 voxels. Age and sex were set as covariates in all the analyses. WM and GM regions corresponding to local maxima within significant clusters were anatomically mapped using FSL standard-space atlases (JHU DTI-based WM atlases for WM; Harvard-Oxford cortical/subcortical structural atlases for GM).

## Results

### General

There was no age heterogeneity among the study groups (*p* = 0.21).

Cognitive impairment was present in two POMS and one AOMS patients.

As expected, disease duration of early adult POMS was higher than AOMS (*p* = 0.01).

### Differences in Macroscopic Brain Damage: Lesions and Volumes

**Figure 1:**
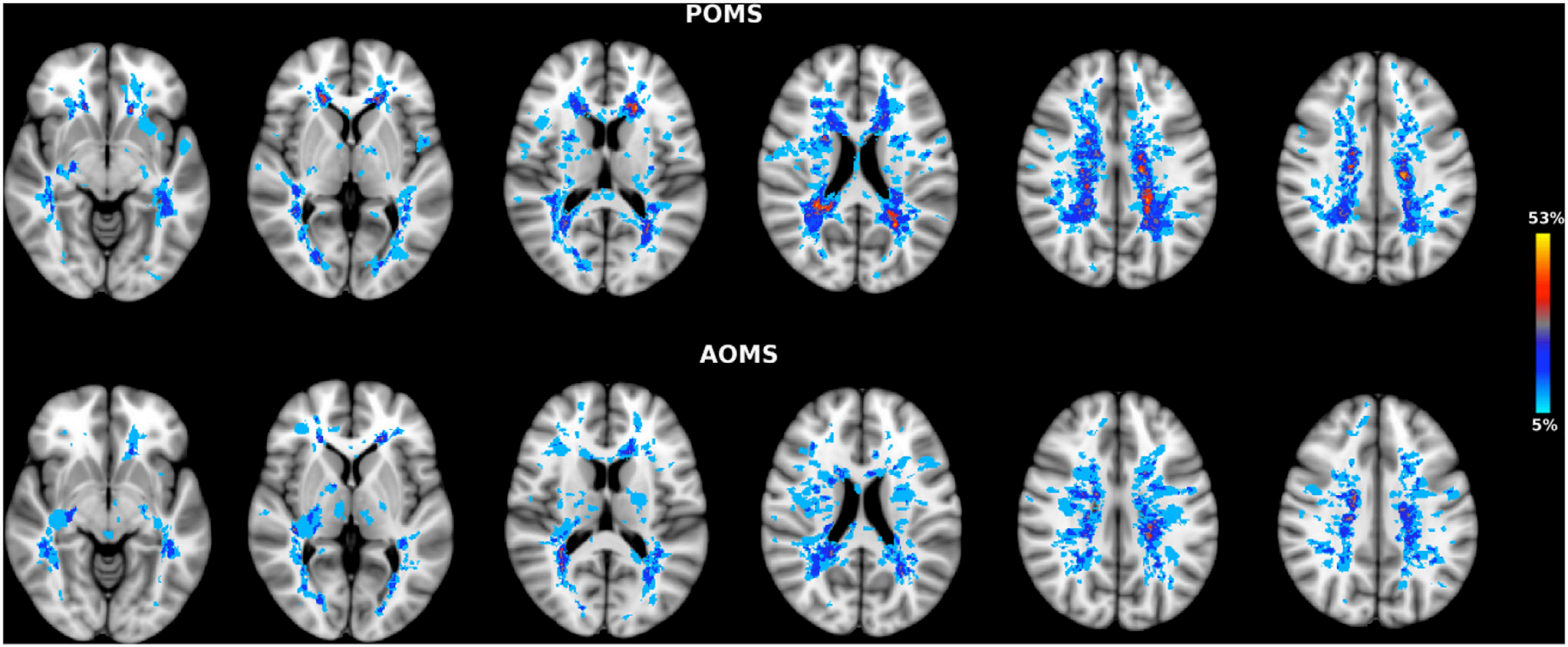
Lesion probability map (LPM) in early adult pediatric-onset multiple sclerosis (POMS) with no or minimal disability (top) and age- and disability-matched adult-onset MS (AOMS) (bottom). Note the similar distribution across brain of T2-lesion LPMs in both patient groups. However, at voxelwise analysis the former group showed higher lesion frequency in two clusters of the posterior corona radiata (see Table 1). The color overlay created on top of the MNI standard brain represents the probability of lesion occurrence (lesion frequency) in a particular spatial location. Images are shown in radiological convention.

Brain WM lesions were present in all patients. LV in early adult POMS was higher, although not significantly, than AOMS (T2-LV: 10.7 ± 12 vs 6.6 ± 4.5 cm^3^, *p* = 0.23). LPMs of the two patient groups showed an overall similar distribution across brain (Figure 1) although, at voxelwise analysis, early adult POMS had higher probability of lesion occurrence (lesion frequency) than age- and disability-matched AOMS in the posterior corona radiata (PCR) (Table 1).

**Table 1:** White matter (WM) regions where lesion frequency of early adult pediatric-onset multiple sclerosis with no or minimal disability was higher than in age- and disability-matched adult-onset MS (*p* < 0.05, corrected).

WM regions (local maxima)	Side	MNI *X*, *Y*, *Z*	Cluster size (voxel count)	*p*-Value
Posterior corona radiata (PCR)	R	26, −40, 27	245	0.02
PCR	R	21, −42, 36	19	0.04

*See text for details*.

Among the various brain volumes assessed (whole brain, WM, GM, and neocortical and deep GM), only deep GM volume was lower than NC (66.2 ± 3.61 cm^3^) in both early adult POMS (61.93 ± 5.45 cm^3^, *p* = 0.04) and AOMS (60.43 ± 6.98 cm^3^, *p* = 0.01), and this was driven by atrophy of the thalamus (NC: 10.68 ± 0.63 cm^3^, early adult POMS: 9.86 ± 1.16 cm^3^, *p* = 0.02, AOMS: 9.41 ± 1.10 cm^3^, *p* = 0.002) and globus pallidus (NC: 2.50 ± 0.25 cm^3^, early adult POMS: 2.25 ± 0.23 cm^3^, *p* = 0.002, AOMS: 2.21 ± 0.24 cm^3^, *p* = 0.004).

No differences in brain volumes were found between early adult POMS and AOMS.

### Voxelwise Differences of AC

Compared with NC, both early adult POMS and AOMS showed altered DTI measures at TBSS analysis (ANOVA) across the whole brain (*p* ≤ 0.01, corrected).

**Figure 2:**
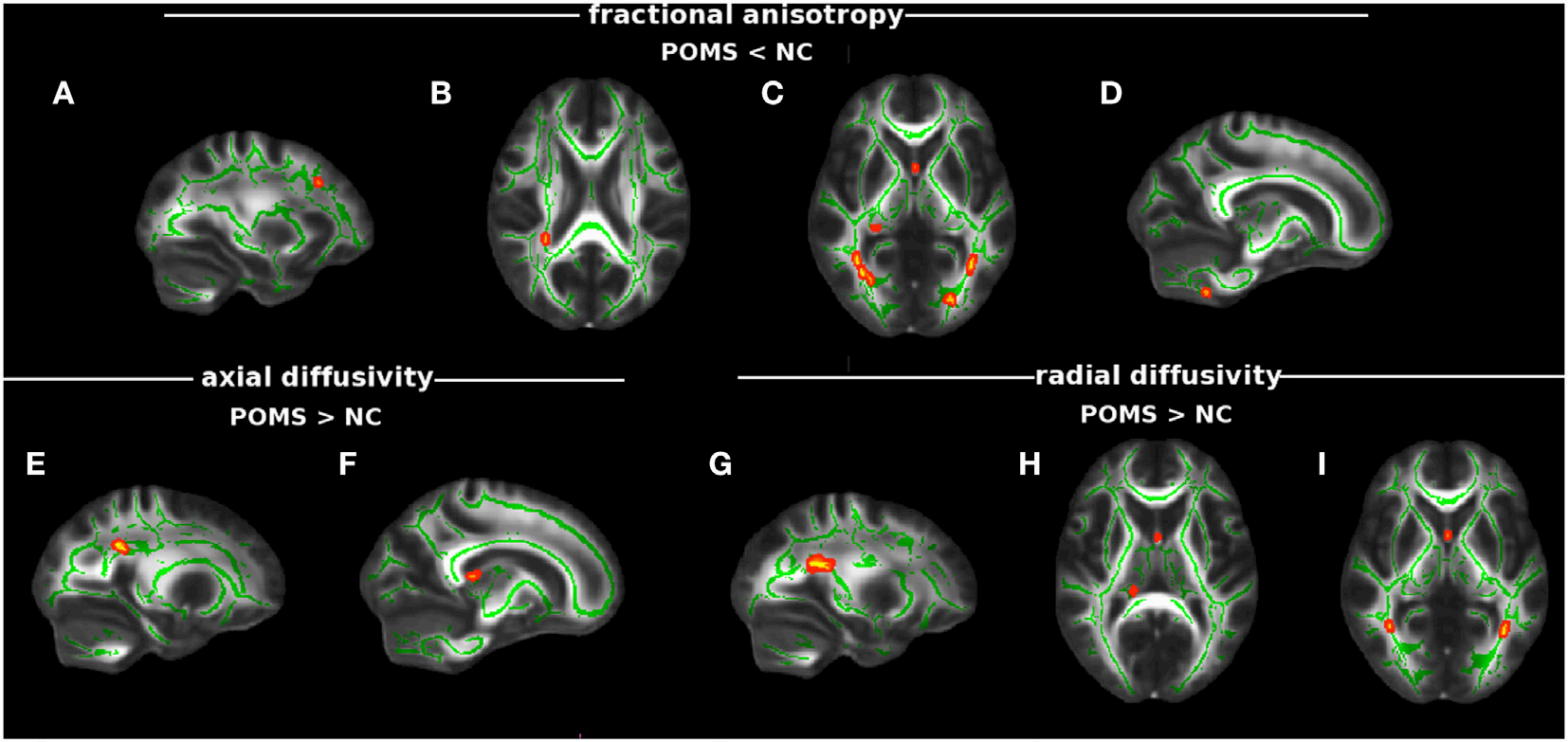
Tract-based spatial statistics analysis of differences in diffusion tensor imaging (DTI) measures between early adult pediatric-onset multiple sclerosis (POMS) with no or minimal disability and normal controls (NC). Red–yellow shows clusters (*p* < 0.01 corrected, thickened for better visibility) of DTI abnormalities in the patient group. In particular, lower fractional anisotropy (FA) was found in white matter (WM) MFG **(A)**, posterior corona radiata (PCR) **(B)**, fornix (Fx), forceps major, inferior fronto-occipital fascicle (IFOF), inferior longitudinal fascicle (ILF), **(C)**, and cerebellum **(D)**; higher axial diffusivity mapped on PCR **(E)** and Fx **(F)**; and higher radial diffusivity was present in PCR **(G)**, Fx **(H)**, IFOF (posterior thalamic radiation), and ILF **(I)**. Green is the mean WM skeleton. Background image, shown in radiological convention, is the FMRIB58_FA standard space.

In particular, POMS showed lower FA (0.46 ± 0.06 vs 0.57 ± 0.03) in clusters of cerebellum (Cb), inferior longitudinal fascicle (ILF), inferior fronto-occipital fascicle (IFOF), forceps major (FM), fornix (Fx), PCR, and middle frontal gyrus WM (Figures 2A–D; Figure S1 in Supplementary Material; Table 2).

**Table 2:** Regions along cerebral white matter (WM) tracts where early adult pediatric-onset multiple sclerosis (POMS) with no or minimal disability showed diffusion tensor imaging (DTI) abnormalities with respect to age- and disability-matched normal controls (NC) and adult-onset MS (AOMS) at tract-based spatial statistics analysis across whole brain (*p* ≤ 0.01, corrected).

WM regions (local maxima)	Side	MNI *X*, *Y*, *Z*	Cluster size (voxel count)	*p*-Value
**POMS vs NC**				
Fractional anisotropy (FA): POMS < NC				
Cerebellum (Cb)	R	15, –51, –43	18	0.01
Inferior longitudinal fascicle (ILF)	R	48, 2, –17	19	0.003
	L	–39, –38, –11	94	<0.001
	R	33, –62, 0	24	0.001
Inferior fronto-occipital fascicle (IFOF)	R	37, –52, –2	65	<0.001
	L	–35, –55, –1	41	0.001
Forceps major	L	–24, –77, 1	17	0.007
Fornix (Fx)	M	0, 4, 6	26	0.007
Posterior corona radiata (PCR)	R	29, –41, 20	15	0.007
WM MFG	L	–29, –22, 33	12	0.005
Axial diffusivity (AD): POMS > NC				
Fx	R	16, –32, 11	11	<0.001
PCR	R	26, –41, 28	17	<0.001
Radial diffusivity (RD): POMS > NC				
ILF	L	–43, –19, –13	89	<0.001
IFOF (posterior thalamic radiation)	L	–35, –56, –3	39	0.005
	R	32, –39, 15	16	0.01
Fx	R	16, –33, 11	28	0.005
PCR	R	29, –36, 19	116	<0.001
**AOMS vs NC**				
FA: AOMS < NC				
Cb		15, –51, –42	16	0.004
ILF	L	–39, –39, –12	94	<0.001
		–42, –26, –15	16	0.004
IFOF	R	37, –52, –2	65	<0.001
	R	33, –59, 0	24	0.006
	L	–36, –53, 1	40	0.001
Fx	M	0, 6, 3	26	0.005
AD: AOMS > NC				
Thalamic WM	R	14, –27, 13	10	0.007
RD: AOMS > NC				
ILF	L	–42, –28, –14	88	0.001
		44, –27, –13		
IFOF	L	–35, –56, –3	39	0.004
Fx	M	0, 8, 0	26	0.004
**POMS vs AOMS**				
AD: POMS > AOMS				
PCR	R	26, –44, 30	17	<0.001
RD: POMS > AOMS				
PCR	R	30, –40, 20	105	<0.001

*For each DTI measure, WM regions are ordered according to increasing Z values. See text for details*.

In addition, early adult POMS patients also showed clusters of altered diffusivities when compared with NC, with higher AD (1.73 ± 0.34 vs 1.35 ± 0.13) in Fx and PCR (Figures 2E,F; Table 2) and higher RD (0.77 ± 0.15 vs 0.57 ± 0.04) in ILF, IFOF (posterior thalamic radiation), Fx, and PCR (Figures 2G–I; Table 2).

**Figure 3:**
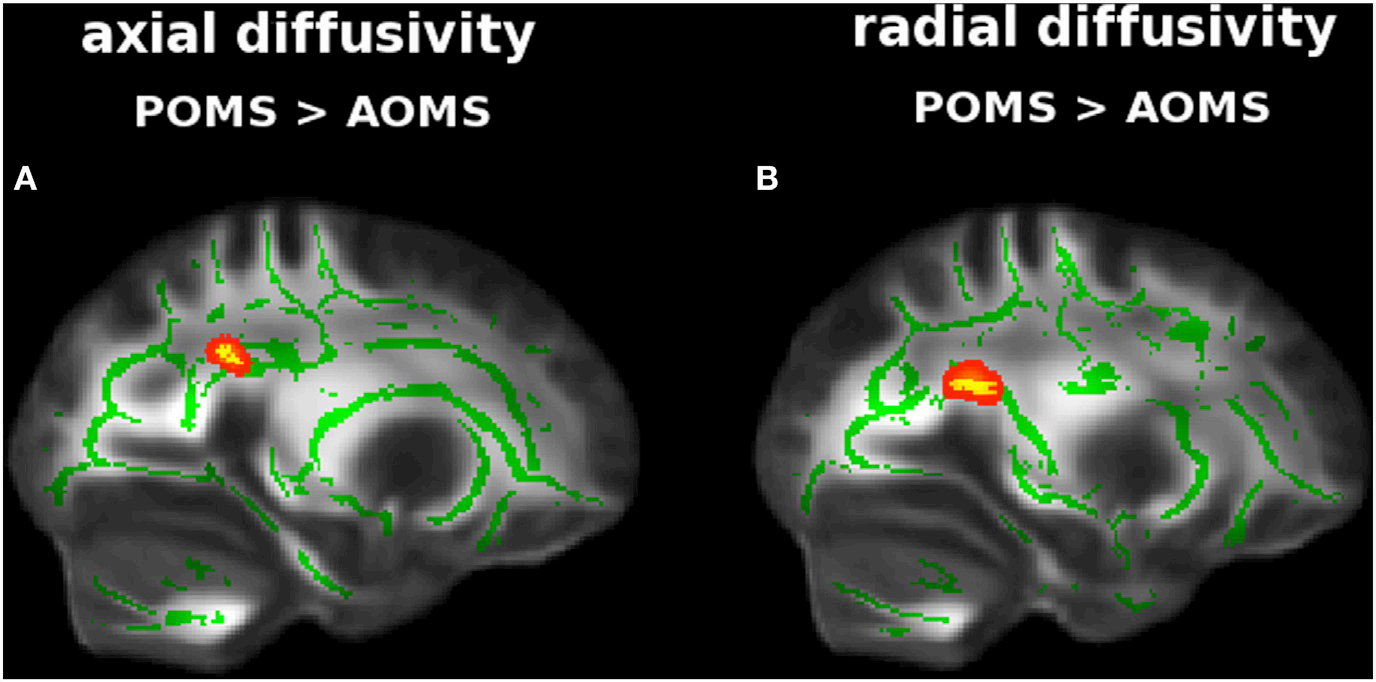
Tract-based spatial statistics analysis of differences in diffusion tensor imaging (DTI) measures between early adult pediatric-onset multiple sclerosis (POMS) with no or minimal disability and age- and disability-matched adult-onset MS (AOMS). Red–yellow shows clusters (*p* < 0.01, corrected, thickened for better visibility) of DTI abnormalities in the former group, with both higher axial diffusivity **(A)** and radial diffusivity **(B)** in posterior corona radiata. Green is the mean white matter “skeleton.” Background image, shown in radiological convention, is the FMRIB58_FA standard space.

Adult-onset MS showed, similarly to early adult POMS, altered DTI measures with respect to NC mapping on various WM tracts. In particular, there was lower FA (0.49 ± 0.04 vs 0.59 ± 0.04) in the Cb, ILF, IFOF (Figure S2 in Supplementary Material); higher AD (2.20 ± 053 vs 1.65 ± 0.31) in the thalamic WM; and higher RD (0.71 ± 0.11 vs 0.53 ± 0.06) along ILF, IFOF, and Fx (Table 2). In addition, early adult POMS showed both higher AD (1.33 ± 0.18 vs 1.22 ± 0.08) and higher RD (0.73 ± 0.17 vs 0.61 ± 0.10) than AOMS in the PCR (Figure 3; Table 2).

### Voxelwise Differences of FC

Across the whole study population 20 functionally relevant RSNs (main networks and subnetworks) were found, including DMN (*n* = 4), executive control network (ECN, *n* = 2), working memory frontoparietal network (*n* = 2), ventral and dorsal attention network (*n* = 4), sensorimotor network (*n* = 3), primary and secondary visual network (VN, *n* = 4), basal ganglia network (BGN, *n* = 1), and cerebellar network (*n* = 1).

**Figure 4:**
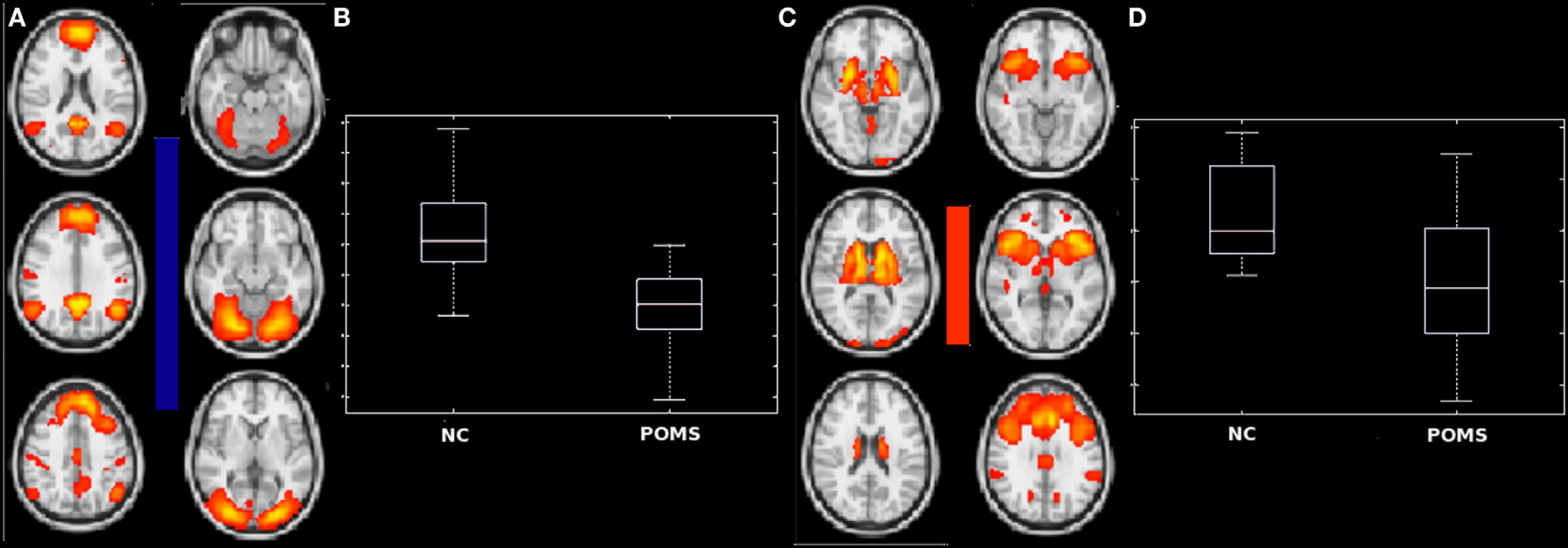
Differences in internetwork functional connectivity between early adult pediatric-onset multiple sclerosis (POMS) with no or minimal disability and normal controls (NC). The negative correlation (blue bar) of anterior default mode network with secondary visual network **(A)** and the positive correlation (red bar) of basal ganglia network and frontal executive control network **(C)** were smaller in POMS than in NC, as showed by the corresponding box-and-whiskers plots of median and range values **(B,D)**. See text for details.

No heterogeneity among the three groups was found in intranetwork FC for any RSN. On the other hand, internetwork FC differences were found. In particular, ANOVA showed two significant differences for full correlation values in early adult POMS and NC, with smaller connection strength in the former group than in the latter: the first (negative correlation) between anterior DMN and secondary VN [median: −4.06 (−0.09 to −7.24) vs −2.6 (1.82 to −4.43), *p* = 0.0016] (Figures 4A,B); the second (positive correlation) between BGN and ECN [median: −0.22 (5.05 to −4.73) vs 2 (0.24–5.91), *p* = 0.01] (Figures 4C,D).

**Figure 5:**
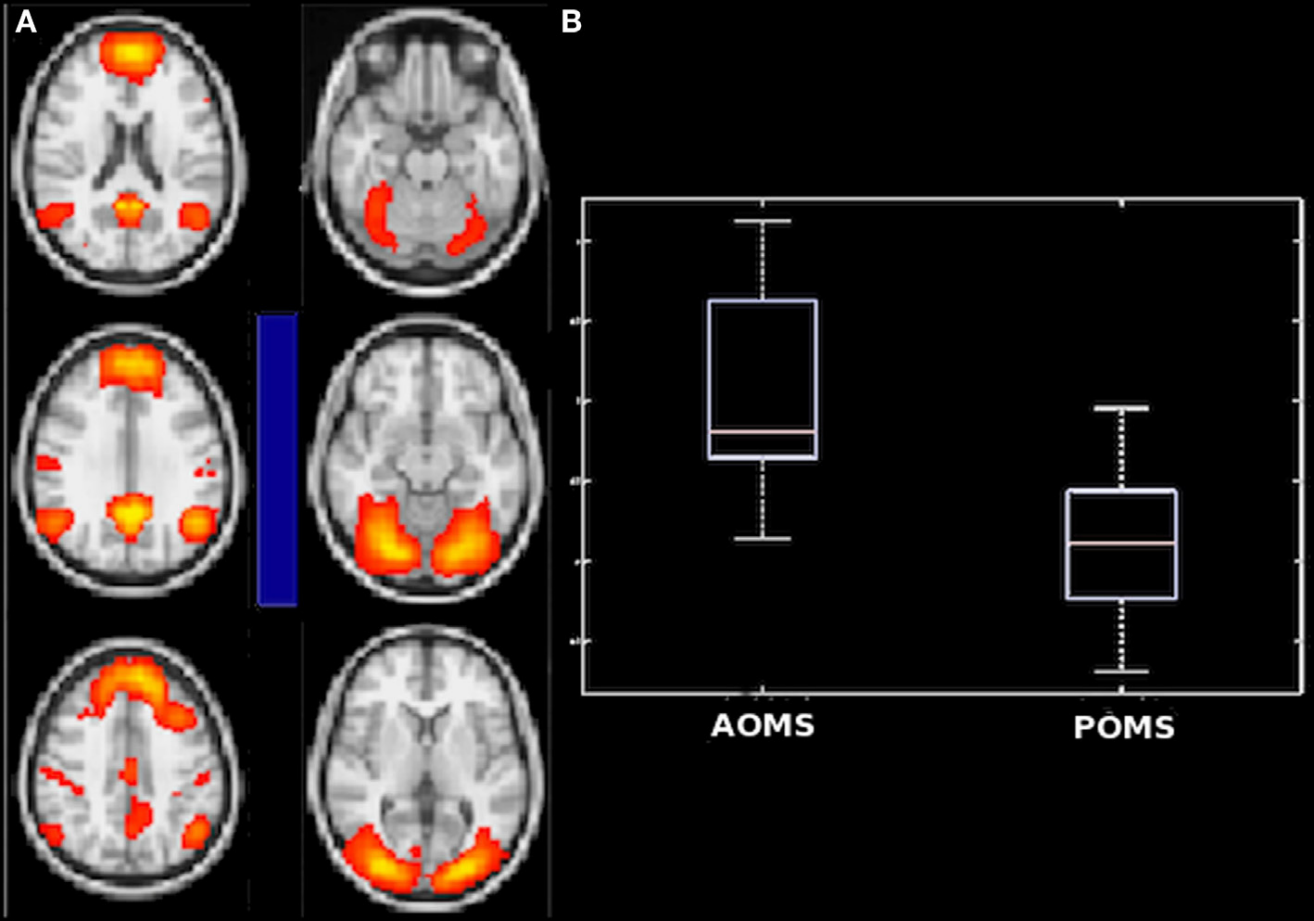
Difference in internetwork functional connectivity between early adult pediatric-onset multiple sclerosis (POMS) with no or minimal disability and age- and disability-matched adult-onset MS (AOMS). Similarly to the comparison with normal controls, the negative correlation (blue bar) of anterior default mode network with secondary visual network **(A)** was smaller in POMS as showed by the corresponding box-and-whiskers plots of median and range values **(B)**. See text for details.

Similarly, young adult POMS and AOMS showed a significant difference for partial correlation in the (negatively correlated) connection strength between anterior DMN and secondary VN, with a decrease in the former group compared with the latter [−0.82 (−0.25 to −1.47) vs −0.22 (−0.66 to 0.65), *p* = 0.0098] (Figures 5A,B). Finally, no significant differences were found between AOMS and NC.

## Discussion

In this study, we found for the first time that POMS patients in their early adulthood and with no or minimal disability showed, despite a similar macroscopic damage (i.e., brain lesion load and deep GM atrophy) to that of AOMS patients with similar age and clinical features, (i) increased lesion frequency and reduced AC along the PCR fibers of the CST, a clinically eloquent tract for physical disability, and (ii) reduced long-range FC involving a relevant hub for cognition such as DMN.

### Structural Damage

In our early adult POMS with no or minimal disability, both higher lesion frequency and altered diffusivities across the whole brain compared with age- and clinically matched AOMS mapped on the PCR. CR is home to fibers of the CST, the most relevant descending projection tract connecting the motor cortex to the spinal cord and its posterior fibers (i.e., PCR) connect to the lower limbs (35). This finding suggests that POMS in the early adulthood and with no or minimal disability show already damage along a WM tract clinically eloquent for ambulation, which represents the major contributor of the disability score (EDSS) in MS. Natural history studies showed that POMS reach irreversible disability and progressive course at a much younger age (i.e., 10 years earlier) than age-matched AOMS patients (8, 9). A recent study identified positive and negative prognostic clinical factors for the occurrence of a second clinical attack in pediatric clinically isolated syndrome and disability worsening in POMS (36). Our results may have a sort of “predictive” meaning for long-term clinical outcome of POMS patients.

Finding signs of structural brain damage in a peculiar MS cohort such as our early adult POMS lead to some general considerations. The well known presence of better short-medium term motor recovery in spite of more inflammation in POMS than in AOMS may be explained not only by “active” mechanisms such as greater adaptive-compensatory plasticity and/or more efficient repair mechanisms but also by an intrinsic stronger “resiliency” due to the less amount of time required for brain reserve utilization. In our study, however, early adult POMS with no or minimal disability showed in the PCR increased lesion frequency and reduced WM tract microstructure. In pathogenic terms, this suggests a greater structural damage and/or lower repair (e.g., remyelination) capacity rather than incomplete myelination, given that by 15 years of age, that is the average onset of our POMS cohort, such process should have been already completed. Our findings on the relevant structural damage of early adult POMS are in line with previous studies using different MRI techniques. Indeed, it was shown in POMS, compared with AOMS, greater T1-LV, a marker of destructive focal pathology, at early stage (14 years of age) but also lower magnetization transfer ratio (MTR), reflecting greater myelin damage, at later adult stage (36 years of age) (37). Then, a significant decrease in MTR recovery was found in acute lesions of POMS during late adolescence (between 16 and 20 years of age) (38). Moreover, altered diffusion measures were found in both visible WM lesions and NAWM of the major WM pathways in adolescent POMS (2, 3). In a more recent study, significant cortical pathology, in terms of a great number of cortical lesions, was detected using ultrahigh-field 7 T MRI in adolescents and young adults with POMS despite relatively brief disease duration and limited physical disability (4).

Evidence exists that POMS and AOMS share similar pathogenic mechanisms in terms of environmental and genetic background (39). However, in POMS immune and nervous systems both undergo continuous changes so that the immunological events of MS can affect the survival and differentiation of oligodendrocyte progenitor cells (i.e., the remyelinating cells), which are in greater amount than adult brain. In agreement with this, compared with AOMS, POMS showed in WM lesions a 50% higher degree of acute axonal damage and higher inflammation (11).

In terms of brain volumes, we found in our study that both early adult MS groups showed deep GM atrophy, especially in the thalamus and globus pallidus compared with NC. On the other hand, no difference in other brain volumes was found between the two MS groups. This is in line with recent studies, where, compared with NC, voxelwise regional GM atrophy was not different between young adult POMS and AOMS (40) and the predominant atrophy in deep GM, including the thalamus, was similar in younger and older onset MS patients matched for short disease duration and clinical status (41).

### Altered FC

We found that our early adult POMS patients with no or minimal disability showed decreased long-range FC. In particular, the connection between DMN and secondary VN was lower than in both NC and age- and disability-matched AOMS. DMN has traditionally showed higher metabolic activity at rest although recent evidence suggests a high intrinsic activity in tasks of memory-based processing. Indeed, a close negative correlation subserving spatial attention has been demonstrated between two core regions of DMN (ventromedial prefrontal cortex and posterior cingulate cortex) and secondary VN (42). Moreover, an increased (i.e., less negative) FC between such two brain networks has been demonstrated after visual learning (43). In line with this, the findings reported here suggest the presence in early adult POMS with no or minimal disability of a selective reduction of long-range FC subserving spatial attention and visual learning not only with respect to NC but also to AOMS.

The second network pair where early adult POMS with no or minimal disability showed decreased FC, but only with respect to NC was BGN and ECN. Executive functions are modulated not only by the classical frontal ECN but also rather by a more widely distributed “executive control circuit” comprising BG, probably involved through learning mechanisms (44, 45). In particular, the caudate nucleus and ventral striatum provide input, respectively, to the frontal cortex and anterior cingulate (46).

In pathogenetic terms, decreased long-range FC in early adult POMS may be justified by exhaustion over time of functional reserve, whose preservation was otherwise demonstrated in adolescent POMS (47) and/or by failure of active compensatory mechanisms due to the accrual of structural burden. Whereas a previous study in adolescent POMS demonstrated a distributed pattern of mainly decreased intranetwork FC and a relative preservation of internetwork FC (6), in this study, we only found evidence of disrupted internetwork FC, suggesting different stage-dependent patterns of FC abnormalities in the POMS brain.

The clinical meaning of mapping decreased long-range FC between pairs of cognition-related brain networks in a POMS group with no or minimal disability is reflected by the fact that usually unfavorable and complex neuropsychological outcomes usually occur in POMS (48) and that a worsening over time of cognition may occur in 75% at 2 years (49) and in 56% at 5 years (10).

Functional connectivity is a broad term used to indicate statistical covariation between timeseries in different brain regions and is considered a proxy for brain region functional interaction. fMRI represents the most commonly used method to consistently map FC at the level of brain networks (50). In recent times, the validity and reproducibility of fMRI turned out to be a highly debated topic (51). The discussion was recently fostered by a study (52) demonstrating that widely used fMRI software packages may have generated over the years a significant amount of false-positive results. However, the same study also pointed out that the only statistical method achieving correct nominal results was the one with minimal statistical assumptions, which is the non-parametric permutation test. Reassuringly, we used such statistical method for all our statistical voxelwise analyses, including fMRI, and thus we are confident about the validity of our results.

The relevance of the current findings stems from the fact that for the first time we selected an early adult POMS cohort who was much younger and with shorter disease duration compared with previous studies in POMS and this allowed us to speculate on the pathogenic mechanisms of MS at a relatively early disease stage.

## Ethics Statement

The study received approval from the local Ethics Committee (Azienda Ospedaliera Universitaria Senese). Informed written consent was obtained from all subjects in accordance with the Declaration of Helsinki before study entry.

## Author Contributions

AG and JZ: acquisition, analysis, and interpretation of data for the work; drafting the work; final approval of the version to be published; and agreement to be accountable for all aspects of the work in ensuring that questions related to the accuracy or integrity of any part of the work are appropriately investigated and resolved. MS, FR, MB, and LN: acquisition and analysis of data for the work; revising the work critically for important intellectual content; final approval of the version to be published; and agreement to be accountable for all aspects of the work in ensuring that questions related to the accuracy or integrity of any part of the work are appropriately investigated and resolved. MM, EP, and BH: acquisition of data for the work; revising the work critically for important intellectual content; final approval of the version to be published; and agreement to be accountable for all aspects of the work in ensuring that questions related to the accuracy or integrity of any part of the work are appropriately investigated and resolved. MA and NS: substantial contributions to the conception and design of the work; revising the work critically for important intellectual content; final approval of the version to be published; and agreement to be accountable for all aspects of the work in ensuring that questions related to the accuracy or integrity of any part of the work are appropriately investigated and resolved.

## Conflict of Interest Statement

The authors report no potential conflicts of interest with respect to the research, authorship, and/or publication of this article.

## Footnotes

^1^http://www.xinapse.com/Manual/.

^2^http://www.fmrib.ox.ac.uk/fsl/.

^3^http://www.nitrc.org/projects/dtiprep/.

^4^https://github.com/rhr-pruim/ICA-AROMA.

^5^http://fsl.fmrib.ox.ac.uk/fsl/fslwiki/MELODIC.

^6^http://fsl.fmrib.ox.ac.uk/fsl/fslwiki/FSLNets.

## Supplementary Material

Figure S1 Illustrative example of the fractional anisotropy (FA) differences between normal controls (NC) and pediatric-onset multiple sclerosis (POMS) rendered in red–yellow on the mean FA image of each group. Crosshairs point at the local maxima of the clusters with lower FA in POMS than in NC obtained with tract-based spatial statistics analysis and mapping on inferior longitudinal fascicle **(A,F)**, inferior fronto-occipital fascicle **(B,G)**, forceps major **(C,H)**, fornix **(D,I)**, and posterior corona radiata **(E,J)**.

Figure S2 Illustrative example of the fractional anisotropy (FA) differences between normal controls (NC) and adult-onset MS (AOMS) rendered in red–yellow on the mean FA image of each group. Crosshairs point at the local maxima of the clusters with lower FA in AOMS than in NC obtained with tract-based spatial statistics analysis and mapping on inferior longitudinal fascicle **(A,D)**, inferior fronto-occipital fascicle **(B,E)**, and fornix **(C,F)**.
