# Supplementary figures and images for: Pronounced Structural and Functional Damage in Early Adult Pediatric-Onset Multiple Sclerosis with No or Minimal Clinical Disability
Source: Front Neurol. 2017 Nov 14;8:608. doi: 10.3389/fneur.2017.00608 (PMC5694464; doi:10.3389/fneur.2017.00608)

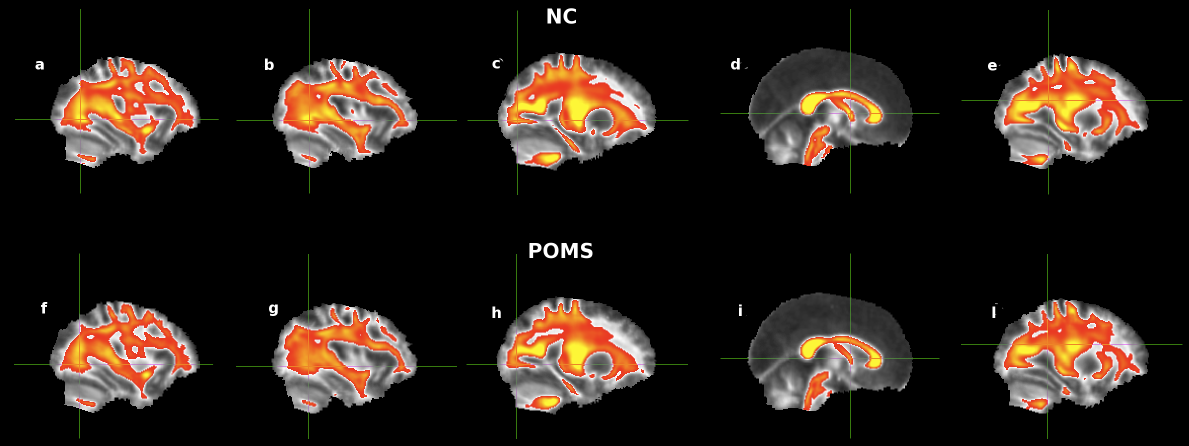

Supplement: Figure S1 — Illustrative example of the fractional anisotropy (FA) differences between normal controls (NC) and pediatric-onset multiple sclerosis (POMS) rendered in red–yellow on the mean FA image of each group. Crosshairs point at the local maxima of the clusters with lower FA in POMS than in NC obtained with tract-based spatial statistics analysis and mapping on inferior longitudinal fascicle (A,F), inferior fronto-occipital fascicle (B,G), forceps major (C,H), fornix (D,I), and posterior corona radiata (E,J). [file image_1.tiff]

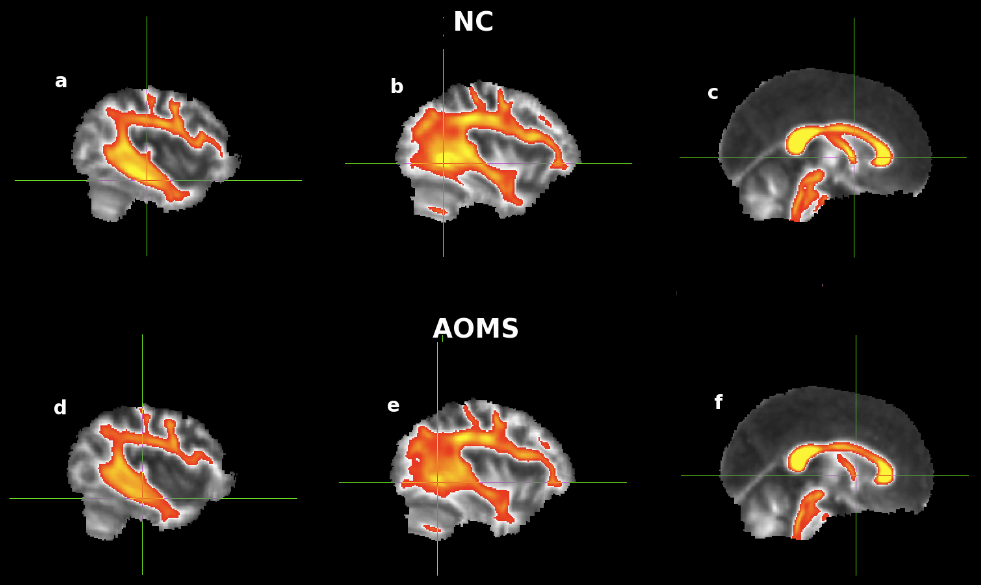

Supplement: Figure S2 — Illustrative example of the fractional anisotropy (FA) differences between normal controls (NC) and adult-onset MS (AOMS) rendered in red–yellow on the mean FA image of each group. Crosshairs point at the local maxima of the clusters with lower FA in AOMS than in NC obtained with tract-based spatial statistics analysis and mapping on inferior longitudinal fascicle (A,D), inferior fronto-occipital fascicle (B,E), and fornix (C,F). [file image_2.tiff]
